# The human microbiome-derived antimicrobial lugdunin self-regulates its biosynthesis by a feed-forward mechanism

**DOI:** 10.1128/mbio.03571-24

**Published:** 2025-03-18

**Authors:** Leonie Reetz, Lukas Schulze, Thales Kronenberger, Khaled A. Selim, Timm Schaefle, Taulant Dema, Alexander Zipperer, Jens Mößner, Antti Poso, Stephanie Grond, Andreas Peschel, Bernhard Krismer

**Affiliations:** 1 Department of Infection Biology, Interfaculty Institute of Microbiology and Infection Medicine, University of Tübingen, Tübingen, Germany; 2 Cluster of Excellence EXC 2124 Controlling Microbes to Fight Infections, Tübingen, Germany; 3 German Center for Infection Research (DZIF), partner site Tübingen, Tübingen, Germany; 4 Department of Pharmaceutical and Medicinal Chemistry and Tuebingen Center for Academic Drug Discovery & Development (TüCAD2), University of Tübingen 9188 , Tübingen, Germany; 5 School of Pharmacy, Faculty of Health Sciences, University of Eastern Finland, Kuopio, Finland; 6 Institute of Phototrophic Microbiology, Heinrich-Heine University Düsseldorf, Düsseldorf, Germany; 7 Institute of Organic Chemistry, University of Tübingen, Tübingen, Germany; Baylor College of Medicine, Houston, Texas, USA

**Keywords:** coagulase-negative Staphylococci, gene regulation, lugdunin, protein modeling, biosynthetic gene cluster

## Abstract

Many human microbiome members inhibit bacterial competitors by production of antimicrobial compounds whose expression needs to be tightly controlled to balance the costs and benefits of compound biosynthesis. The nasal commensal *Staphylococcus lugdunensis* outcompetes *Staphylococcus aureus* using the antimicrobial lugdunin. The lugdunin biosynthetic gene cluster (BGC) encodes two potential regulators whose roles have remained unknown. Deletion of the regulator genes *lugR* or *lugJ* led to increased lugdunin production and/or immunity. While LugR was found to repress the transcription of the biosynthetic *lugRABCTDZ* operon, LugJ repressed the *lugIEFGH* export and immunity genes. Both regulators bound to different inverted repeats in the controlled promoter regions. Notably, both repressors were released from cognate promoters to allow transcription upon addition of exogenous lugdunin. Even minor structural changes disabled lugdunin derivatives to induce expression of its BGC, which is consistent with inferior binding to the predicted LugR and LugJ binding pockets. Thus, lugdunin controls its own biosynthesis through a feed-forward mechanism probably to avoid futile production.

**IMPORTANCE:**

Biosynthetic gene clusters (BGCs) are usually tightly controlled to avoid production of costly goods at inappropriate time points or unfavorable conditions. However, in most cases, the regulatory signals of these clusters have remained unknown. Frequently, quorum sensing or two-component regulatory systems are involved in BGC expression control. This study elucidates the sophisticated regulation of lugdunin biosynthesis and secretion via two independent regulators, LugR and LugJ. Although belonging to different families of repressors, both directly interact with the antimicrobial lugdunin and thereby enhance biosynthesis and secretion in a feed forward-like mechanism.

## INTRODUCTION

The fitness of bacteria in complex microbial communities is governed by different types of interactions, including mutualistic and antagonistic mechanisms. Many bacteria produce antimicrobial compounds, bacteriocins, and related molecules to inhibit microbial competitors. Bacteriocin production is a highly variable and dynamic trait, which is often used by bacteria in nutrient-poor environments (1). Since the production of antimicrobial molecules and establishment of resistance in the producer requires substantial energy, bacteria usually regulate the expression of biosynthetic gene clusters (BGCs) for antimicrobials according to environmental cues (2, 3). The signals and signal transduction mechanisms governing the production of bacteriocin-related antimicrobials have remained unknown for most of the BGCs and are a neglected field of research.

In contrast to primary metabolites, bacteriocins and other secondary metabolites are not essential for the survival of bacteria but contribute to the fitness in specific ecological niches (4, 5). Bacterial secondary metabolites are, for example, important for bacterial intra- and inter-species competitions or cooperation, as well as for the evasion of host defense mechanisms in host-associated habitats (6, 7). Bacteriocins and other bacterial antimicrobial molecules are also of potential importance as alternatives to antibiotics for the prevention or treatment of antibiotic-resistant infections, which are on the rise (5, 8, 9). In addition to the specific antimicrobial molecules, commensal bacteria producing such molecules are also considered for probiotic microbiome-targeted interventions (10). In nutrient-poor natural habitats, such as the human nares, members of the microbiome need to compete effectively to prevail using a large variety of bacteriocin-like molecules (4, 11, 12). The most frequent members of the adult human nasal microbiome belong to the genera *Staphylococcus*, *Corynebacterium*, *Cutibacterium*, *Dolosigranulum*, as well as *Moraxella* and *Finegoldia* (12–14). Population-based studies have shown that about 20% of human individuals permanently carry the aggressive pathogen, *Staphylococcus aureus*, in their nose (15, 16). This carriage is a major risk factor for invasive *S. aureus* infections. *S. aureus*-non-carriers are probably protected from *S. aureus* because their nasal microbiomes include *S. aureus*-eradicating commensals (14). We are only beginning to understand which commensals are beneficial against *S. aureus* colonization and which types of antimicrobials they use. One previously identified member of the nasal microbiome is *Staphylococcus lugdunensis*, which outcompetes *S. aureus* by the production of the cyclic peptide antimicrobial, lugdunin (17–19).

Lugdunin is the founding member of a new class of cyclic thiazolidine peptides. It acts as a protonophore against gram-positive bacteria and is especially effective against *S. aureus*, which occupies the same ecological niche as *S. lugdunensis* (20). In fact, the presence of lugdunin-producing *S. lugdunensis* in the human nose reduces the risk of *S. aureus* colonization by about sixfold (19). Although the relative abundance of *S. lugdunensis* is usually low compared to *S. aureus*, the production of lugdunin and its synergistic effect with human-derived antimicrobial peptides are sufficient to eradicate *S. aureus* from the human nose (21). Which environmental conditions may induce lugdunin production and how expression of the lugdunin BGC could be improved remain as important research topics.

We report here that the lugdunin BGC encodes the two repressors, LugJ and LugR, to control the major transcriptional units of the BGC. The product lugdunin itself serves as an inducer of the expression of the lugdunin biosynthesis and transport genes in a feed forward-like fashion. The two repressors are unrelated, but both are released from their operator motifs upon binding of lugdunin. Structural models identify potential distinct binding sites within LugJ and LugR dimers. These findings will help to engineer lugdunin-producing bacteria to ensure high-enough lugdunin production for long-term protection against *S. aureus* colonization.

## RESULTS

### LugR and LugJ are regulators of the lugdunin BGC

**Fig 1:**
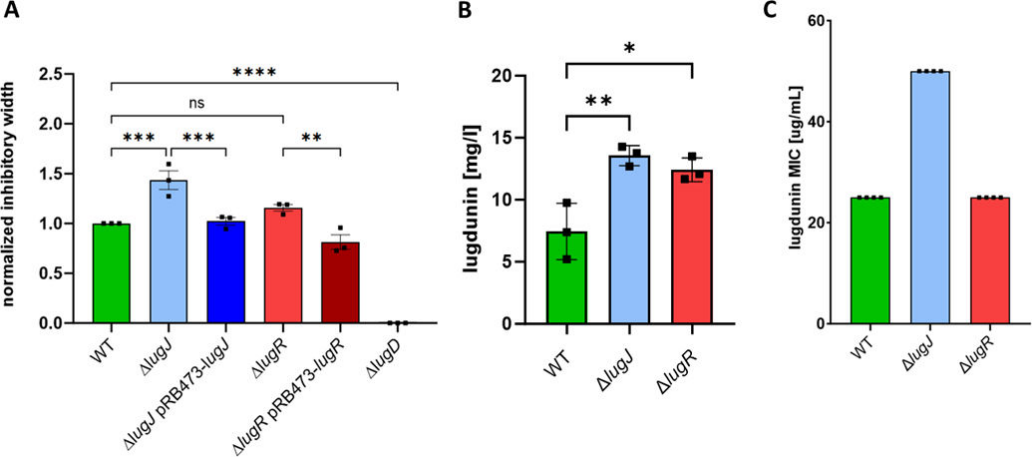
(**A**) Both *S. lugdunensis* IVK28 Δ*lugR* and Δ*lugJ* elicit larger inhibition zones on *S. aureus* USA300 LAC lawns than the wild type. (**B**) *S. lugdunensis* Δ*lugR* and Δ*lugJ* show 1.67- and 1.82-fold higher lugdunin production in the liquid culture, respectively. (**C**) While *S. lugdunensis* IVK28 Δ*lugR* has the same lugdunin MIC as the wild type (wt), *S. lugdunensis* Δ*lugJ* tolerates ca. two-fold higher lugdunin concentrations. Data points represent mean values ± standard deviation of three independent experiments. All data were analyzed using one-way analysis of variance. ns = not significant, *P < 0.05, **P < 0.01, ***P < 0.001, and ****P < 0.0001.

The lugdunin gene cluster is composed of 13 genes with multiple functions in lugdunin synthesis, export, immunity, and potentially regulation. Those genes for lugdunin biosynthesis (*lugRABCTDZ*), as well as transport and immunity (*lugIEFGH*), have previously been characterized (19, 22). Additionally, two putative regulators, LugR and LugJ, whose roles have remained unknown, are encoded in the BGC. LugR belongs to the TetR-like regulators (23). Its gene is located directly upstream of the biosynthetic genes, most likely together forming the *lugRABCTDZ* transcriptional unit. Upstream of the immunity and transport genes, transcribed in the opposite orientation, the second putative regulator, LugJ, a winged helix–turn–helix-type regulator, is encoded (24). To analyze putative functions of LugR and LugJ as regulators and identify their impact on lugdunin biosynthesis, secretion, and immunity, clean deletion mutants for both genes were constructed by allelic recombination. Additionally, both deletions were complemented by plasmid-encoded copies of *lugJ* and *lugR*. As shown in Fig. 1A, both mutants, Δ*lugR* and Δ*lugJ*, showed a stronger inhibition of the *S. aureus* USA300 LAC indicator strain than the wild type, indicating a significant impact of the two genes on lugdunin biosynthesis or export. While Δ*lugR* showed a 16% increased width of the inhibition zone, Δ*lugJ* exhibited a 45% larger inhibitory distance. Complementation of the deletions resulted in wild type-level inhibition for pRB473-*lugJ* (2% increase) and even less than wild type-level inhibition for pRB473-*lugR* [19% reduction (Fig. 1A)]. Quantification of lugdunin production in liquid culture by wild-type and mutant strains revealed that Δ*lugR* and Δ*lugJ* secreted 1.67- and 1.82-fold more lugdunin than the wild type, respectively (Fig. 1B). To elucidate if the regulators also impact the lugdunin immunity of the strains, susceptibilities of the strains to lugdunin were compared. While Δ*lugR* showed no change in susceptibility, the Δ*lugJ* mutant exhibited a two-fold increase in the lugdunin minimal inhibitory concentration (MIC) (Fig. 1C) compared to the parental strain. These data suggested a critical function of LugR and LugJ in lugdunin biosynthesis or transport and of LugJ in lugdunin immunity.

### LugR and LugJ negatively regulate lugdunin biosynthesis and export

**Fig 2:**
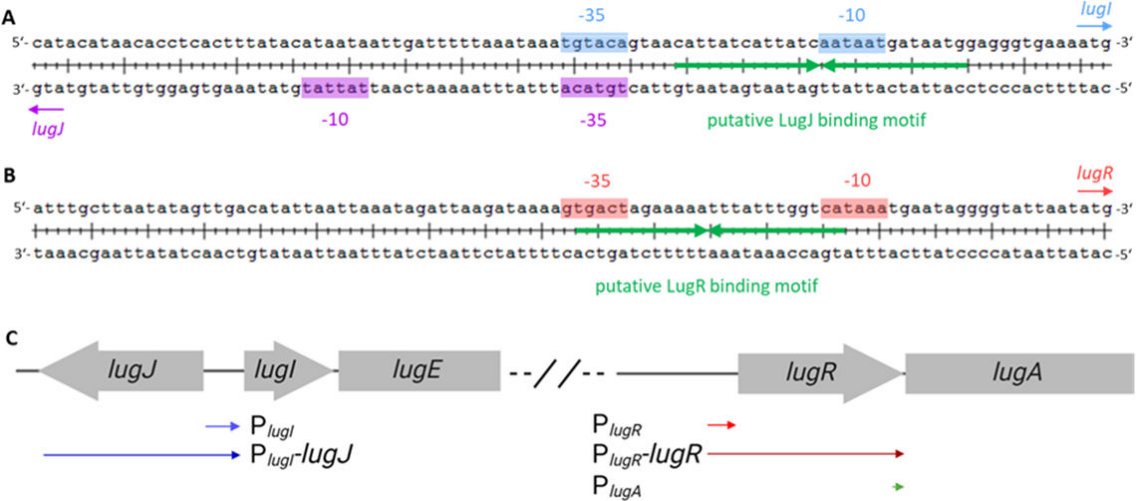
Genetic organization of the promoter regions of the *S. lugdunensis* IVK28 lugdunin BGC. (**A**) Intergenic region between *lugJ* and *lugI*. Highlighted are the start codons of *lugJ* (purple arrow) and *lugI* (blue arrow) as well as the −10 and −35 promotor motifs. A palindrome that could be a putative regulator binding motif is shown in green. (**B**) Promoter region of *lugR* with the predicted −10 and −35 motifs upstream of the *lugR* start codon (red arrow). A palindromic putative regulator binding motif is shown in green. (**C**) Schematic overview of the genomic operon of the lugdunin BGC. Shown below are the genetic constructs for the YFP reporter system. Arrows indicate the promoter orientation upstream of *yfp* on the pCG725 plasmid. *P* signifies the putative promoter sequence of the gene in subscript, which was cloned either alone (no regulator) or followed by the gene encoding the putative regulator LugJ or LugR, as indicated.

To elucidate the precise roles of the two regulators, the putative promoter regions of the transcription units *lugIEFGH* and *lugRABCTDZ* were examined. The very short 90-base pair (bp) intergenic region between *lugJ* and *lugI* (positions 823,452–823,541 of GenBank accession number CP063143) should contain two promoters in opposing orientations to drive expression of *lugJ* or *lugIEFGH*. However, they appear to share the −35 region 5′-TGTACA-3′, which differs in two positions from the consensus sequence 5′-TTGACA-3′ and represents a palindrome that could serve both directions (Fig. 2A). The putative −10 promoter regions occur at the appropriate distance for both directions, 5′-TATTAT-3′ as the −10 region of *lugJ* and 5′-AATAAT-3′ as the −10 region of *lugIEFGH*, both differing by one nucleotide from the consensus sequence 5′-TATAAT-3′. Analysis of the sequence further revealed a large, nearly perfect 26 bp palindrome (5′-**CATTATCATTAT**CA**ATAATGATAATG**-3′) between the −35 region and the *lugI* ribosomal binding site, which could serve as a repressor binding site (Fig. 2A).

The 227 bp intergenic region between the 3′-end of *lugH* and the 5′-start of *lugR* was also analyzed for putative promoter and operator sequences (positions 827,680–827,906 of GenBank accession number CP063143). Immediately upstream of *lugR,* putative −35 (5′-GTGACT-3′) and −10 (5′-CATAAA-3′) regions could be identified, interestingly forming part of a 24 bp palindromic sequence (5′-g**TGAC**T**A**G**A**A**AAATTT**A**T**T**T**G**GTCA**taaa-3′), which might also serve as a repressor binding motif (Fig. 2B).

**Fig 3:**
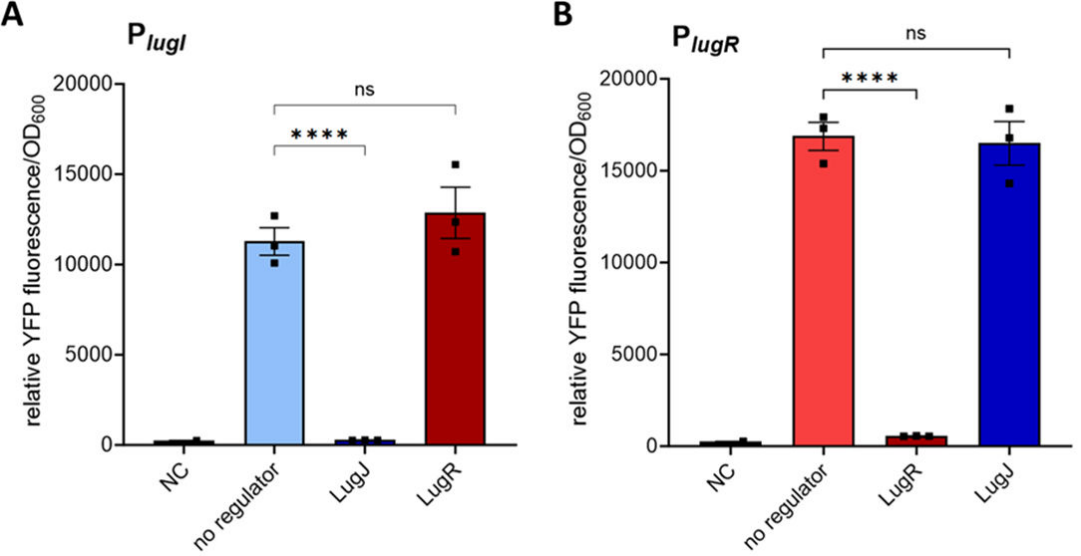
Effect of LugJ and LugR on the activity of the putative promoters as determined by YFP reporter assays in *S. aureus*. Promoter strength measured by endpoint YFP fluorescence activity of *S. aureus* PS187 ΔΔ strains carrying the *yfp* reporter constructs indicated in Fig. 2C. The fluorescence was normalized to OD_600_ = 1 after 24 h of growth. (**A**) Strength of P*_lugI_* in absence (no regulator) or presence of either of the putative regulators LugJ or LugR. (**B**) Promoter strength of P*_lugR_* in the absence (no regulator) or presence of either of the putative regulators LugJ or LugR. NC: negative control. Data represent mean values ± standard error of the mean of three independent biological replicates. All data were analyzed using one-way analysis of variance. ns = not significant, *P < 0.05, **P < 0.01, ***P < 0.001, and ****P < 0.0001.

To confirm that the noncoding DNA regions between the *lug* operons contain promoters that are indeed potentially regulated by LugR or LugJ, the putative promoter regions were cloned in front of a yellow-fluorescent protein (YFP) reporter gene and used for transformation of the restriction–modification deficient *S. aureus* strain PS187 Δ*hsdR* Δ*sauUSI* (25) (further referred to as *S. aureus* PS187 ΔΔ). The putative promoter sequences were cloned either alone or together with one of the two regulator genes. A schematic overview of the constructs that were used for the transformation is given in Fig. 2C. Indeed, the putative *lugI* promoter led to a strong expression of the YFP in *S. aureus* PS187 ΔΔ. Its activity was reduced to background level (2–3% of maximum expression) in the presence of the regulator gene *lugJ* but not *lugR* (Fig. 3A). Likewise, the putative *lugR* promoter also led to a strong YFP expression, which was downregulated by the presence of *lugR* (3–4% of maximum expression) but not of *lugJ* (Fig. 3B).

**Fig 4:**
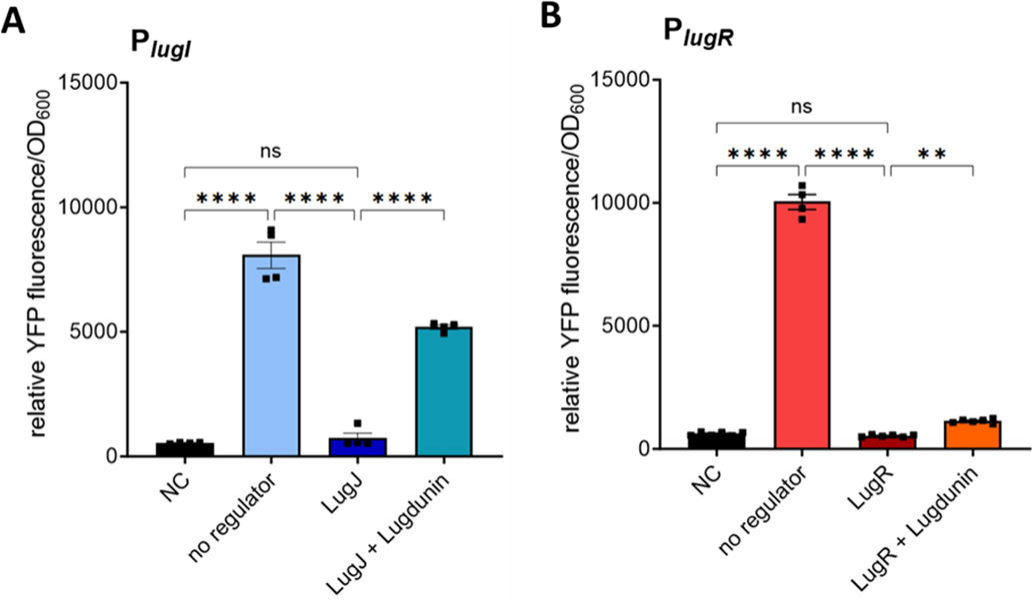
Effect of LugJ and LugR on the activity of the putative promoters as determined by YFP reporter assays in *S. lugdunensis* IVK28 Δ*lugJ* (P*_lugI_*) and Δ*lugR* (P*_lugR_*). The promoter strength measured by endpoint YFP fluorescence activity of *S. lugdunensis* IVK28 carrying the *yfp* reporter constructs indicated in Fig. 2C. The fluorescence was normalized to OD_600_ = 1 after 24 h of growth. (**A**) Strength of P*_lugI_* in absence (no regulator) or presence of the putative regulator LugJ and induction by lugdunin (2 µg/mL). (**B**) Promoter strength of P*_lugR_* in the absence (no regulator) or presence of the putative regulator LugR and induction by lugdunin (2 µg/mL). NC: negative control. Data represent mean values ± standard error of the mean of three independent biological replicates. All data were analyzed using one-way analysis of variance. ns = not significant, *P < 0.05, **P < 0.01, ***P < 0.001, and ****P < 0.0001.

To analyze promoter regulation in *S. lugdunensis* IVK28, the P*_lugI_* and P*_lugI_–lugJ* constructs were transduced into the *S. lugdunensis* Δ*lugJ* mutant to avoid regulatory effects of the chromosomal *lugJ* copy. Likewise, the P*_lugR_* and P*_lugR_-lugR* constructs were analyzed in the Δ*lugR* mutant. Similar to *S. aureus*, the *lugI* and *lugR* promoters without their cognate repressors caused a strong YFP fluorescence. In the presence of the regulatory genes, fluorescence was reduced to the background level of the negative control (Fig. 4A and B).

These results indicated that LugJ and LugR both act as repressors, albeit for different promoters. LugJ regulates the transporter operon *lugIEFGH*, whereas LugR has an auto-regulatory function controlling its own expression and that of the presumably co-transcribed biosynthesis genes *lugABCTDZ*. To prove that the promoter of *lugR* is, in fact, responsible for the expression of the biosynthesis genes, which are presumed to be cotranscribed with *lugR*, and exclude that the intergenic region between *lugR* and *lugA* contains an internal promoter, two independent analyses were performed. First, an RT-PCR with a *lugR*-specific forward primer and a *lugA*-specific reverse primer was performed on DNAse I-digested RNA isolated from *S. lugdunensis* IVK28, as well as on reverse-transcribed cDNA. A PCR product of 660 bp was only detected with cDNA as the template, which confirms that the biosynthetic genes *lugA* and *lugR* are located on the same transcriptional unit. The RNA did not result in a PCR product, thereby confirming that the RNA preparation was free of chromosomal DNA (Fig. S1A). Second, the DNA sequence upstream of *lugA* was cloned in front of the YFP reporter gene in a similar way as the *lugR* promoter upstream of *lugR*. While the *lugR* promoter yielded robust reporter gene activity, the DNA fragment upstream of *lugA* did not, thereby confirming that lugA is not expressed from own promoter (Fig. S1B).

### Native lugdunin controls its own biosynthesis and transport

Since LugR and LugJ are repressors, we analyzed the *S. lugdunensis* IVK28 culture for putative inducers, which would de-repress the promoters in a *lugR* or *lugJ*-dependent fashion. To this end, whole *S. lugdunensis* IVK28 cultures were extracted with 1-butanol, and subfractions obtained by high-performance liquid chromatography (HPLC) were analyzed for influences on the *lugI* or *lugR* promoter activities in the presence of LugJ or LugR. A strong de-repression of both promoters by distinct HPLC fractions obtained from the *S. lugdunensis* IVK28 WT strain was observed (Fig. S3A). Analysis of these fractions by HPLC–coupled mass spectrometry (HPLC–MS) revealed the presence of lugdunin as a common constituent of the inducing fractions, suggesting that lugdunin itself might be an inducer of *lugI* and *lugR* promoter activities. As a control, a culture of the lugdunin non-producing mutant *S. lugdunensis* IVK28 Δ*lugD* (19) was extracted and microfractionated as described above, but none of its fractions showed de-repression capabilities (Fig. S3B). This finding supports the hypothesis that lugdunin induces its own biosynthesis and transport.

Assuming that lugdunin itself might activate the genes of the lugdunin BGC by interaction with the regulatory proteins, the specificity of such interactions was analyzed in the heterologous reporter gene expression system using native lugdunin (1) or synthetic lugdunin variants. To this end, three previously published derivatives (structures in Fig. S4), enantio-lugdunin (2), which has the opposing conformation but the same antimicrobial activity as native lugdunin, fourfold less antimicrobially active 2-Ala-lugdunin (3), and antimicrobially inactive N-methylthiazolidine-lugdunin (4) (20, 26) were selected.

**Fig 5:**
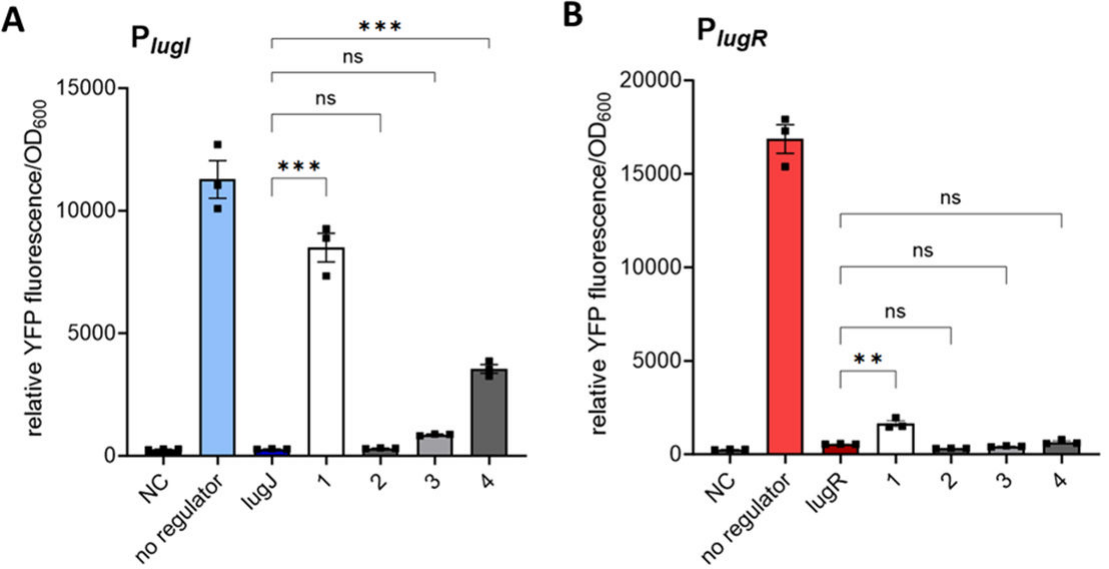
Specificity of inducers for the promoter activity of P*_lugI_* and P*_lugR_* in the YFP reporter assay. Endpoint measurements of YFP fluorescence in strains carrying the reporter construct P*_lugI_–lugJ* (**A**) and P*_lugR_–lugR* (**B**) after induction with 2 µg/mL lugdunin (1) or the derivatives enantio-lugdunin (2), 2-Ala-lugdunin (3), and N-methylthiazolidine-lugdunin (4). The measured YFP intensities were correlated to the respective OD_600_ after 24 h of growth at 37°C. NC; negative control. Data represent mean values ± standard error of the mean of three independent biological replicates. All data were analyzed using one-way analysis of variance. ns = not significant, *P < 0.05, **P < 0.01, and ***P < 0.001.

To minimize antimicrobial effects on the growth of the heterologous reporter strain *S. aureus* PS187 ∆∆*,* inhibitory concentrations of lugdunin of 5 µg/mL (22) and its derivatives were avoided and limited to sub-inhibitory 2 µg/mL. This concentration of native lugdunin was sufficient to de-repress the *lugI* promoter in the presence of LugJ to approximately 75% of the unregulated promoter activity in the absence of *lugJ* (Fig. 5A), thereby confirming that it is indeed lugdunin, which induces *lugI* transcription. The same lugdunin concentration also increased the LugR-repressed *lugR* promoter activity, albeit only to 10% of the promoter activity measured in the absence of the regulator gene (Fig. 5B). De-repression was not observed by enantio-lugdunin (2). 2-Ala-lugdunin (3) increased the YFP expression slightly from 3 to 8% for the LugJ-repressed P*_lugI_* promoter, but no increase was detected for the P*_lugR_–lugR* construct. Only the addition of N-methylthiazolidine-lugdunin (4) resulted in significant induction of P*_lugI_*–lugJ, leading to 32% of the fluorescence of the unrepressed promoter. In contrast, no significant increase could be detected for the P*_lugR_–lugR* construct with compound 4 (Fig. 5A and B). This pattern indicates a high specificity of the regulators for the natural inducer lugdunin, which is obviously unrelated to the antimicrobial activity. The addition of 2 µg/mL lugdunin also led to a partial de-repression of P*_lugI_* (64% of the reporter activity of the free promoter) and P*_lugR_* (11% of free promoter) in *S. lugdunensis* (Fig. 4A and B).

### LugJ and LugR act as homodimers on the respective promoters

**Fig 6:**
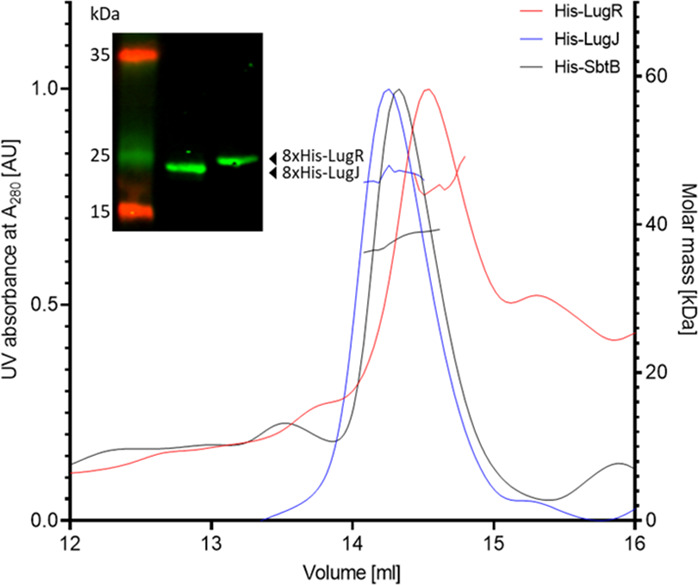
Characterization of native His-LugR and His-LugJ oligomerization states. SEC–MALS analysis of the purified proteins revealed that both exist in a dimeric state with a calculated mass of approximately 46.8 (His-LugR) and 47.3 kDa (His-LugJ). The theoretical size of 26.3 (His-LugR) and 24 kDa (His-LugJ) could be detected in western blot analysis (inset). Trimeric His-SbtB [*P*(II)-like signaling protein] eluted around the same retention time and with a molar mass of approximately 40 kDa was used as a positive control.

To further examine the structure and function of LugR and LugJ, both proteins were expressed with an N-terminal 8×His-tag in *Escherichia coli* Rosetta-gami. After purification, proteins were used for size-exclusion chromatography coupled to multiangle light scatter (SEC–MALS) analysis to assess the oligomerization state of the proteins (27). Proteins in the eluted peaks were collected and precipitated by the addition of trichloroacetic acid (TCA) and analyzed by SDS-PAGE, followed by western blot analysis. Retention volumes of 14.2 and 14.5 mL were determined for 8×His-LugJ and 8×His-LugR, respectively (Fig. 6), corresponding to masses of 47.3 (8×-His-LugJ) and 46.8 kDa (8×His-LugR) for the native proteins as indicated by MALS analysis. However, the calculated monomeric masses of 24 and 26.3 kDa were detected by western blot of denaturing SDS-PAGE gels using an anti-His antibody, indicating that both proteins in their native state associate as dimers.

**Fig 7:**
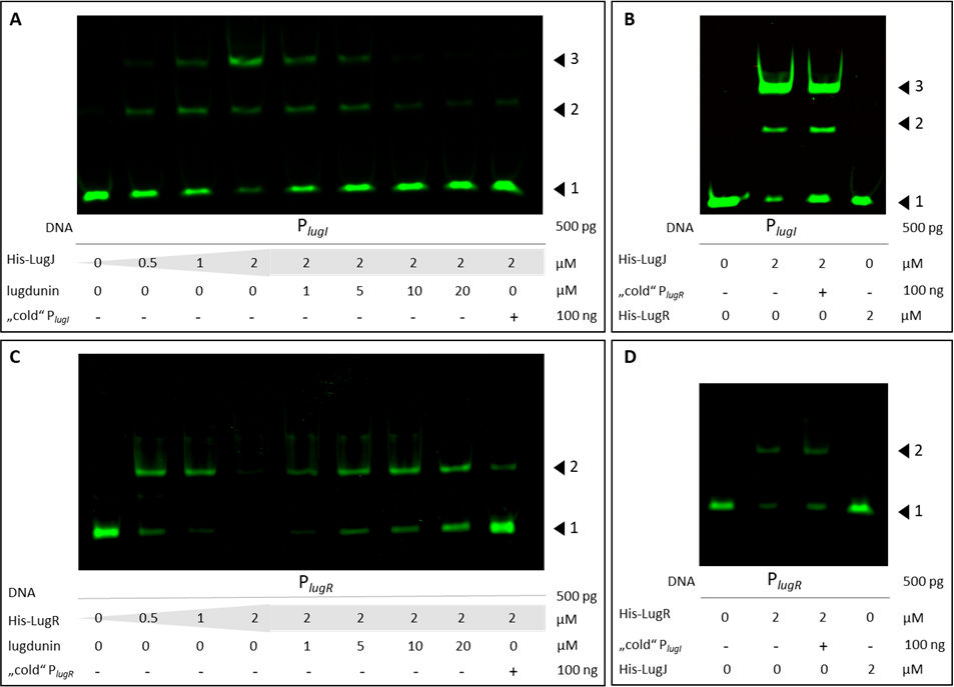
Interaction of promoter sequences and regulators verified by EMSA analysis. DY-781-labeled DNA fragments, proteins, and lugdunin were incubated in EMSA buffer and subjected to native PAGE. (**A–D**) 500 pg of the DY-781-labeled P*_lugI_* or P*_lugR_* PCR fragment was used for interaction studies with His-LugJ or His-LugR, respectively. (**A**) Increasing amounts of His-LugJ (0 to 2 µM) were added, leading to a shift of P*_lugI_*. Addition of lugdunin (1 to 20 µM) abrogated this shift just as the addition of 100 ng unlabeled (“cold”) P*_lugI_*. (**B**) In a control experiment, the shift of P*_lugI_* was not affected by the addition of “cold” P*_lugR_*, and His-LugR was not able to shift the P*_lugI_* fragment. (**C**) Increasing amounts of His-LugR (0 to 2 µM) were added, leading to a shift of P*_lugR_*. Addition of lugdunin (1 to 20 µM) abrogated this shift just as the addition of 100 ng unlabeled (“cold”) P*_lugR_*. (**D**) In a control experiment, the shift of P*_lugR_* was not affected by the addition of “cold” P*_lugI_*, and His-LugJ was not able to shift the P*_lugR_* fragment. Black arrows indicate the positions of the shifted promoter fragments (1: unbound promoter fragment; 2: shifted DNA–protein complex; 3: double shifted DNA–protein complex).

To confirm that LugR and LugJ impact the expression of lugdunin biosynthesis and transport genes by directly binding to the respective promoters, the purified proteins were combined with promoter DNA fragments encompassing the *lugI* or *lugR* promoters, which were then analyzed by electrophoretic mobility shift assays (EMSA). Both LugJ and LugR bound only the cognate promoter sequences they had been found to regulate in the reporter gene assay in a concentration-dependent manner (Fig. 7A and C), whereas none of the proteins bound to the other promoter (Fig. 7B and D). To analyze if the palindromes in the promoter sequences represent indeed the regulator binding motifs, the unlabeled (“cold”) palindromes, including 7–11 bp at the 5′- and 3′-ends, were added to the EMSA reaction mixes. As demonstrated in Fig. S2A and B, only the native palindromes abrogated the shift of LugR or LugJ by direct competition. To evaluate the specificity of this competitive binding, two “scrambled” versions of the palindromes were tested, at which “scram1” contains the two palindrome halves in a swapped order, and “scram2” contains individual nucleotide exchanges (see Table 1). For His-LugR, the two scrambled palindrome versions still had some capacity to reduce specific binding by the native palindrome, albeit with a reduced efficiency (Fig. S2A). In contrast, none of the scrambled versions of the LugJ-binding palindrome was able to interfere with the specific binding of the native palindrome sequence (Fig. S2B). Addition of increasing concentrations of lugdunin to regulator-bound promoter fragments led to a dose-dependent release of free DNA. Only a molar excess of lugdunin compared to regulator concentrations resulted in an almost complete detachment of DNA from the proteins (Fig. 7A and C), confirming that lugdunin has a direct impact on the capacity of the regulators to bind to their specific recognition sequences.

**TABLE 1:** Primers and plasmids used in this study with restriction sites underlined

Primer or plasmid	Sequence or description	Assignment
Primers		
lugH SacI	TTGGAGCTCCCTTTATTACAACGTTCATAG	*lugR* knockout
lugH Acc65I u	TTCGGTACCTATTCATTTATGACCAAATAA	*lugR* knockout
lugA Acc65I d	GACGGTACCGTTGATAGGATTTTTGAATAAG	*lugR* knockout
lugA BglII	TTTAGATCTGCCAGTCAATTCAAACTTTGC	*lugR* knockout
lugJ K.O. upstream SacI	ATCGAGCTCACTGGTAACAAAGAACAAG	*lugJ* knockout
lugJ K.O. upstream Acc65I	GATGGTACCTACTAGCAAAAGTTAAAAC	*lugJ* knockout
lugJ K.O. downstream Acc65I	TTAAATGGTACCTCAATAATGATAATGGAG	*lugJ* knockout
lugJ K.O. downstream BglII	ATTAGATCTGGCTTTCGACTGGTTACA	*lugJ* knockout
lugI Prom Sph	ATTGGCATGCATAATAGAAAGTCCTTCTGG	P_*lugI*_
lugI Prom Sal	CACCGTCGACATTATCATTATTGATAATGATAA	P_*lugI*_ and P_*lugI*_-*lugJ*
lugJ Prom Sph	TTCATTTTCGCATGCCATTATCATTATTG	P_*lugJ*_
lugJ Prom Sal	AACAGTCGACTTTATACATAATAATTGATTT	P_*lugJ*_
lugJ Sph	CCAGCATGCCTAGGATTAACTTGAGAGG	P_*lugI*_-*lugJ*
lugR Prom Sph	TAAAGCATGCAATAAGAAATACAATGTAAATG	P_*lugR*_ and P_*lugR*_-*lugR*
lugR Prom Sal	AATACCCCTAGTCGACTATGACCAAATAAAT	P_*lugR*_
lugRA Prom Sal	ACCTCCGTCGACTAAAACTTATTCAAAAATCC	P_*lugR*_-*lugR*
lugR_prom_EcoRV	TAAAGATATCAATAAGAAATACAATGTAAATG	P_*lugI*-_*lugR*
lugR_prom_PstI	ACCTCCCTGCAGTAAAACTTATTCAAAAATCC	P_*lugI*_-*lugR*
lugJ_prom_EcoRV	TTTTGATATCTCATTATTGATAATGAT	P_*lugR*_-*lugJ*
lugJ_prom_BamHI	TAGCAGGATCCCCTAGGATTAACTT	P_*lugR*_-*lugJ*
lugA_prom_SphI	AATGCATGCCTTAGGATATATCTTTACTCC	P_*lugA*_
lugA_prom_SalI	ATTGGTCGACTTTAACTAAAACTTATTCAAAAATCC	P_*lugA*_
lugR forward	ttgtgaaaagcattggtcaaaatacgg	RT-PCR
lugA reverse	caccatgtttccgtatataacgatctagtc	RT-PCR
His-lugJ for	GTCGCGGATCCATGGAATTCAGTT	His-*lugJ*
His-lugJ rev	GGTGCTCGAGACATTATGGAATAAT	His-*lugJ*
His-lugR for	GTCGCGGATCCATGGCATTCTCTA	His-*lugR*
His-lugR rev	GGTGCTCGAGACTTATTCAAAAATCCT	His-*lugR*
8xHis for	GGCAGCAGCCATCATCATCATCATCATCATCATAGCAGCGGCCTGGTGCCGC	8×His-tag
8xHis rev	GCGGCACCAGGCCGCTGCTATGATGATGATGATGATGATGATGGCTGCTGCC	8×His-tag
YFP_up	GGTAAGTTTTCCGTATGTTGCATC	Sequencing upstream YFP
pCG725_down	CCTTTTGCTCACATGTTCTTTCC	Sequencing upstream YFP
pCG725_Seq_ insert_for	GTATTATATTTTGTATTATCGTTGA	Sequencing downstream YFP
pCG725_Seq_ insert_rev	TTCTGTTAACTTACTAACTC	Sequencing downstream YFP
IR800 lugI_Prom_for	(DY-781) - ACATAACACCTCACTTTATACATAA	*lugR* promotor sequence for EMSA
IR800 lugI_Prom_rev	(DY-781) - CATTTTCACCCTCCATTATCATTATTG	*lugR* promotor sequence for EMSA
IR800_ lugR_Prom_for	(DY-781) - CAATGTAAATGTAGTATAAAATCCAC	*lugI* promotor sequence for EMSA
IR800_ lugR_Prom_rev	(DY-781) - ATTAATACCCCTATTCATTTATGACC	*lugI* promotor sequence for EMSA
Pal_lugR__native_fwd	gataaaagtgactagaaaaatttatttggtcataaatgaatag	Native LugR palindrome
Pal_lugR__native_rev	ctattcatttatgaccaaataaatttttctagtcacttttatc	Native LugR palindrome
Pal_lugR__scram1_fwd	gataaaagtttatttggtcatgactagaaaaataaatgaatag	Scrambled LugR palindrome 1
Pal_lugR__scram1_rev	ctattcatttatttttctagtcatgaccaaataaacttttatc	Scrambled LugR palindrome 1
Pal_lugR__scram2_fwd	gataaaagtTaAtTgTaTaGCtTaAtAgAtTataaatgaatag	Scrambled LugR palindrome 2
Pal_lugR__scram2_rev	ctattcatttatTaAcTaTtTaGCtTtAcAaAtTacttttatc	Scrambled LugR palindrome 2
Pal_lugI__native_fwd	gtacagtaacattatcattatcaataatgataatggagggtg	Native LugI palindrome
Pal_lugI__native_rev	caccctccattatcattattgataatgataatgttactgtac	Native LugI palindrome
Pal_lugI__scram1_fwd	gtacagtaaataatgataatgcacattatcattatgagggtg	Scrambled LugI palindrome 1
Pal_lugI__scram1_rev	caccctcataatgataatgtgcattatcattatttactgtac	Scrambled LugI palindrome 1
Pal_lugI__scram2_fwd	gtacagtaaAaGtTtAaGtTtcaaAaGtTaAaTtAgagggtg	Scrambled LugI palindrome 2
Pal_lugI__scram2_rev	caccctcAaGtTtAaGtTttgaAaGtTaAaTtAttactgtac	Scrambled LugI palindrome 2
Plasmids		
pCG725	YFP reporter assay	
pET-28a	Protein expression	
pRB473	Complementation plasmid	
pBASE6	Construction of knockouts	

These results indicate that both regulators, LugR and LugJ, probably act as homodimers on distinct lugdunin BGC promoters, where specific palindromic sequences are recognized and bound by the regulators. Addition of lugdunin, the biosynthetic product of the BGC, abrogates the binding and results in increased expression of the biosynthetic genes *lugABCTDZ*, as well as the transporter genes *lugIEFGH*. The observation of a second shifted band with increasing concentrations of LugJ result from either an unrecognized second “low-affinity” binding site in the promoter or more likely by the formation of a LugJ tetramer at higher protein concentrations.

### Lugdunin can stably bind to predicted binding sites in LugR and LugJ structure models

**Fig 8:**
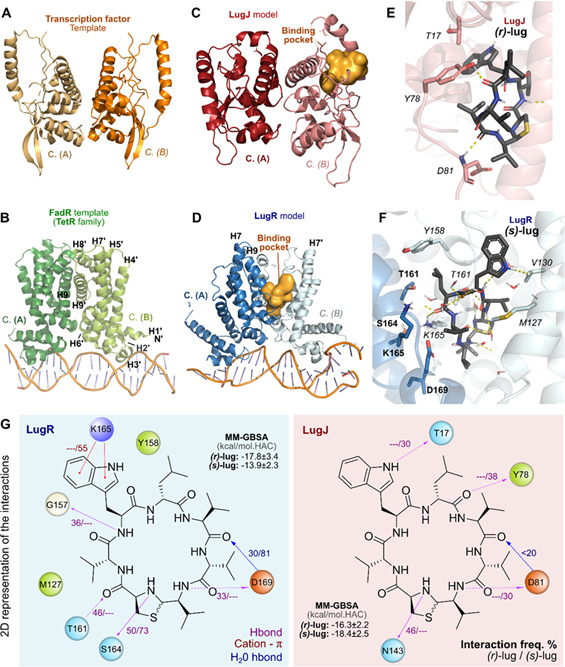
(*r,s*)-Lugdunin can stably bind to the predicted binding sites in LugR and LugJ. Templates utilized in model generation for LugJ (**A**) and FadR (**B**). Final model and highlighted druggable binding pockets (orange) for LugJ (**C**) and LugR (**D**). Three-dimensional representation of the lugdunin binding mode for LugJ (**E**) and LugR (**F**). Polar contacts are highlighted as yellow dashed lines. Bold and italic labels represent amino acids from different chains performing interactions. These structures represent the most populated cluster in the molecular dynamics simulations. (**G**) Two-dimensional schematic representation of lugdunin interactions along the simulations (at least 1 µs, i.e., 5 × 200 ns) in both configurations (*r*) and (*s*). Binding energy is estimated using the molecular mechanics–generalized Born surface area method and displayed as the mean of the simulation and standard deviation both normalized by the number of heavy atoms in the ligand.

We generated LugJ and LugR monomeric structure models using AlphaFold and inferred homodimeric states of the two proteins based on selected homologues. The LugJ dimeric structure was modeled based on a putative transcription factor from *Kribbella flavida* (PDB ID: 4G6Q), which was the highest-ranking structure in terms of resolution and secondary structure similarity (Fig. 8A). LugR was modeled after the homodimeric apostructure of FadR (PDB ID: 5GPC), an acyl–CoA or fatty acid-binding member of the TetR regulator family, bound to dsDNA from *Bacillus halodurans* (Fig. 8B) (28). The inferred dimeric structures of LugJ and LugR were congruent with those of other dimeric DNA-binding proteins.

We identified potentially drug-binding (druggable) binding pockets (DScores > 0.9, anything above 0.8 is considered druggable) in each monomer of LugJ (Fig. 8C) within the alpha-helix bundle (i.e., the pocket composed of H4′, H5′, H7′, H8′, and H9′, with a DScore 1.12). In contrast, the druggable pocket of LugR, whose homodimeric fold is Ω-shaped, resides in the middle of the dimerization interface (Fig. 8D). The predicted pocket of LugR superimposes with the tetracycline binding site of TetR orthologues (29). Interestingly, a comparison of different structures of the LugR-related FadR indicated that ligand binding prevents the interaction with DNA.

Since lugdunin exists in a racemic mixture of the thiazolidine’s chiral center (20), docking and further calculations were performed for both (*r*)- and (*s*)-lugdunin. Selected docking poses were submitted to short molecular dynamics simulations (see supplemental material section on the extra methods) to evaluate the stability of the proposed interactions and calculate lugdunin’s binding energy. Interestingly, (*s*)-lugdunin displays a more stable predicted binding mode on LugJ (Fig. 8E and G), with lower predicted binding energy and more abundant stable polar interactions than (*r*)-lugdunin. In contrast, (*r*)-lugdunin seems to be the preferred ligand of LugR (Fig. 8F and G). Our models propose potential binding modes for all combinations of lugdunin configurations and protein binding (Supporting information, Fig. S5A). However, the key interactions between lugdunin and LugJ or LugR are rather transient, which is reflected in the overall binding energy along the simulations.

Single-point energy calculations generated with the inactive or less active lugdunin variants 2, 3, and 4 (Fig. S4) suggest a much worse binding energy for all (*r*)-configurations of the (*r*)-lugdunin analogues *r*-2, -3, and -4 for LugR. LugJ binding of the various *s*-isomers also worsened compared to its affinity for unaltered lugdunin (Fig. S5B). This increase in the binding energies is likely due to distinct reasons, such as the lost hydrophobic contacts between LugR’s Met127 and the methyl group from variant 3 (an isopropyl moiety in the original lugdunin) or loss of hydrogen bond interactions with Ser164 (LugR) and Asn143 (LugJ) due to the amino-methylation present in variant 4.

## DISCUSSION

We demonstrate that the two major transcription units of the lugdunin BGC are regulated by repressors that bind to the BGC promoter regions. The operons for lugdunin biosynthesis and export and immunity are controlled by different repressors (i.e., LugR and LugJ, respectively), of which LugR also controls its own expression. LugJ did not affect transcription of the *lugR* promoter. Likewise, LugR did not repress the *lugI* promoter. The two repressors belong to different families of DNA-binding proteins and do not share any similarity, but they respond to the same inducer, the BGC-encoded protonophor lugdunin. It seems odd that the BGC uses two different regulators for the same purpose. Nevertheless, based on reporter expression data and modeled binding pockets, LugR and LugJ appear to have different binding modes for lugdunin. De-repression of the *lugIEFGH* promoter by particularly low lugdunin amounts may be of importance since immunity gene expression should be secured, even at moderate lugdunin concentrations, while maximum expression of biosynthesis genes may be required at later stages when full-level immunity has been established.

**Fig 9:**
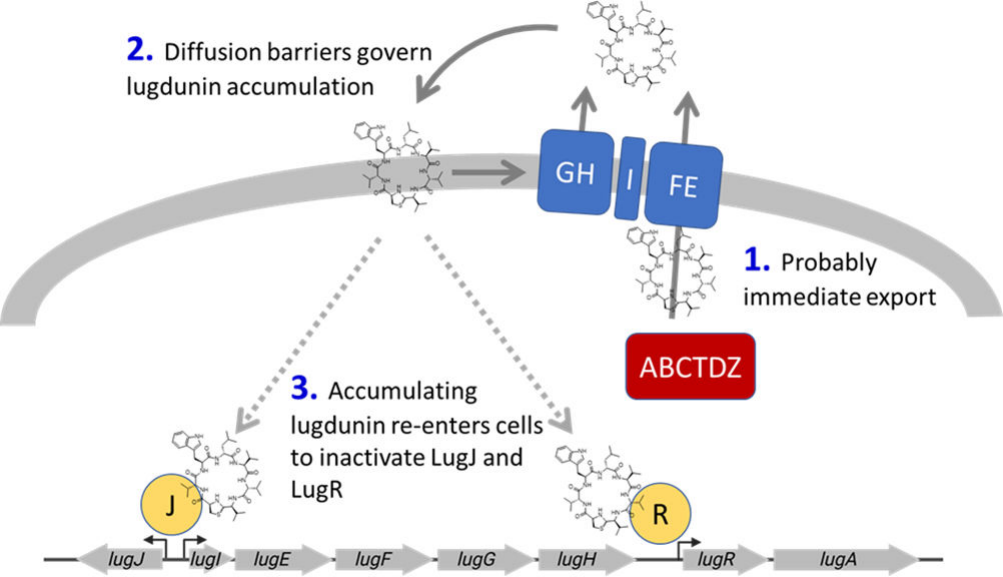
LugJ and LugR act as homodimeric repressors of the *lugIEFGH* and *lugRABCTDZ* operons, respectively. Lugdunin acts as an inducer to release the repressors from the promoters, leading to the expression of the lugdunin biosynthetic and transport genes. This effect seems to be highly specific, as the lugdunin enantiomer and other derivatives cannot or can only slightly induce the system. For simplicity, only part of the lugdunin BGC is shown.

It was surprising to find that lugdunin does not inhibit its own biosynthesis genes as often found in metabolic regulatory feed-back loops, but that lugdunin activates its own expression. Such a concept of “feed-forward” regulation or autoinduction (30) seems to make sense for *S. lugdunensis* considering the varying habitats of the bacteria and the fact that lugdunin production is only reasonable for the producer in environments where lugdunin can accumulate to reach concentrations that allow inhibition of bacterial competitors. In habitats with continuous liquid secretion, lugdunin will be strongly diluted, and its costly biosynthesis will be useless for *S. lugdunensis*. However, in habitats with limited diffusion, such as epithelial micro-niches and hair follicles, lugdunin may easily reach sufficient concentrations to inhibit competitors, such as *S. aureus*. Instead of harnessing a typical *Staphylococcus* house-keeping quorum sensing system, such as *agr* (31), the lugdunin BGC employs its product lugdunin both as an autoinducer and as an antimicrobial effector (Fig. 9). Although lugdunin has a high tendency to integrate into membranes because it acts as a protonophore (20), the lugdunin BGC does not encode a trans-membrane sensory protein, which could report lugdunin amounts in the cytoplasmic membrane of the producer. We demonstrate that LugJ and LugR both bind lugdunin directly, which suggests that lugdunin that accumulates extracellularly also diffuses back to the cytoplasmic membrane of the producer cells and may enter the cytoplasm to de-repress the *lugIEFGH* and *lugRABCTDZ* operons. This regulation principle can also limit excessive lugdunin production because lugdunin increases expression of the exporter proteins, which will reduce intracellular lugdunin concentrations, eventually restoring the repressor function of LugR and LugJ to silence both *lug* promoters and cease lugdunin production (Fig. 9).

Recently, we have shown that the transporters LugIEFGH confer resistance to native lugdunin in *S. aureus* but not, or only to a lesser extent, to lugdunin derivatives, such as enantio-lugdunin (2) or 6-Trp-lugdunin, even though these congeners have antimicrobial activity (22). Here, we show that antimicrobially active enantio-lugdunin and 2-Ala-lugdunin (3) are also no ligands for LugJ and LugR and are unable to de-repress the *lug* promoters. Only the antibacterially inactive N-methylthiazolidine-lugdunin (4) exhibited some inducing capacity on LugJ, indicating that not only transport and immunity but also induction of the BGC-encoded genes is highly specific for natural lugdunin.

While the addition of lugdunin led to a strong de-repression of the *lugIEFGH* promoter (approximately 75% of the unregulated promoter), the same lugdunin concentration led to only 10% activity of the unregulated *lugRABCTDZ* operon in our heterologous reporter gene system. Highly similar values were obtained for the natural producer, *S. lugdunensis* IVK28. One explanation for the weak de-repression of the *lugR* promoter could be that LugR is less sensitive to lugdunin de-repression than LugJ. The calculations suggest the highest affinity for (*r*)-lugdunin in the binding pocket of the LugR dimer contact zone when the repressor is DNA-bound. As soon as the (*r*)-lugdunin–LugR complex dissociates from the DNA, the calculated free-binding energy for (*r*)-lugdunin changes from −17.8 to −9.3 kcal/mol, which would result in the dissociation of (*r*)-lugdunin and the binding back of the repressor to the DNA. In contrast, LugJ has a high affinity for (*s*)-lugunin of −18.4 kcal/mol, even when it is not bound to DNA. The structure of the LugR orthologue FadR has previously been determined both in its active dimer apo form and in its inactive monomeric state co-crystallized with palmitic acid. Structural comparison between these different forms confirms that ligand binding prevents DNA interaction (28), suggesting that LugR might also lose its DNA-binding capacity upon lugdunin binding. The used lugdunin concentration was, therefore, probably not sufficient to achieve a stronger de-repression of the *lugRABCTDZ* promoter, but we had to keep it at subinhibitory amounts. An alternative explanation lies in the genetic organization since LugR represses its own transcription. Induction of transcription necessarily increases the concentration of LugR, which will again repress transcription in the absence of sufficient amounts of the inducer. These possible explanations might reflect a very efficient mechanism to ensure that the lugdunin concentration never exceeds a specific critical concentration since also the producer, *S. lugdunensis* IVK28, is not fully resistant against its own product (22).

Regulatory proteins are found in most of the BGCs for antimicrobial compounds, but, in most of the cases, the regulatory signals have remained unknown. Actinobacteria, the typical and best-studied antibiotic producers, appear to link antibiotic gene expression to global cellular circuits in incompletely understood ways. Quorum sensing-linked LuxR-type regulators appear to play a major role in actinobacterial antibiotic producers (2, 32). In some cases, intracellular second messengers, such as ppGpp, are involved (33). It remains to be investigated how the lugdunin gene expression may be linked to global regulatory mechanisms of *S. lugdunensis*. Feed-forward regulation has also been reported for the actinobacterial lanthipeptides microbisporicin (34) and planosporicin (35), the angucycline jadomycin B (36), the anthracycline daunorubicine (37), and the pyrrolamide congocidine (38). Moreover, the characterization of antimicrobial BGCs in the Bacillota (previously Firmicutes) has led to additional examples of auto-inducing antimicrobial compounds. The lanthipeptides nisin and subtilin, for instance, induce their own expression via the NisRK and SpaRK two-component regulator system, respectively (39, 40). Thus, autoinduction of antibiotic synthesis may be more common than currently acknowledged.

Our study might be the first to confirm direct binding of an antimicrobial product to a regulatory protein governing expression of an antibiotic BGC, which will help to elucidate general regulatory mechanisms guiding the production of antimicrobial compounds. Our finding will also help to assess under which environmental conditions lugdunin may be effectively produced to support the fitness of *S. lugdunensis* in competition with other bacteria.

## MATERIALS AND METHODS

### Bacterial strains and culture conditions

*Escherichia coli* DC10B and *E. coli* Rosetta-gami 2(DE3) were used for plasmid construction and protein expression, respectively. *S. aureus* PS187 Δ*hsdR* Δ*sauUSI* (25) was used as a heterologous host for reporter gene expression. *S. lugdunensis* IVK28 wild type and Δ*lugD* mutant were used for the extraction of putative activators of lugdunin BGC transcription. *S. lugdunensis* IVK28 Δ*lugJ* and Δ*lugR* were used for reporter gene expression studies. All strains were cultivated at 37°C under continuous shaking. *E. coli* strains were incubated in lysogeny broth (Lennox, LB [Carl Roth GmbH, Germany]), *S. lugdunensis* and *S. aureus* in basic medium (BM: 1% soy peptone [Organotechnie SAS, France], 0.5% yeast extract [Deutsche Hefewerke GmbH, Germany], 0.5% NaCl, 0.1% glucose, and 0.1% K_2_HPO_4_). Appropriate selection was achieved using antibiotics ampicillin at 100 µg mL^−1^ or kanamycin at 25 µg mL^−1^ for *E. coli* and chloramphenicol 10 µg mL^−1^ for *S. aureus*. MIC assays were performed as described before (22).

### RNA isolation and reverse transcription PCR

*S. lugdunensis* IVK28 was cultured overnight before the strain was inoculated to an OD_600_ of 0.1 in fresh BM medium under continuous shaking at 120 rpm at 37°C for 6 h. Then, 500 µL of culture was pelleted by centrifugation at 11,000 × *g* for 1 min. The pellet was resuspended in 1 mL of TRIzol and stored at – 80°C until further use. For RNA isolation, the sample was thawed, and 200 µL of chloroform was added and mixed by shaking before incubation at room temperature (RT) for 3 min. After phase separation by centrifugation at 12,000 × *g* for 15 min at 4°C, the upper phase was carefully pipetted and added to 500 µL of isopropanol. After centrifugation at 12,000 × *g* for 30 min at 4°C, the supernatant was discarded, and the RNA pellet was washed with 500 µL of 70% ethanol. The pellet was dried for 60 min at RT to evaporate the residual ethanol from the previous step. Afterwards, the pellet was dissolved in 100 µL of RNase-free water. RNA was purified using the NucleoSpin RNA Clean-up Kit (Macherey-Nagel, Düren, Germany). To remove potential DNA contaminations, RNA was treated with 1U DNAse I (Thermo Scientific) for 1 h according to the manufacturer’s instructions before the enzyme was heat inactivated (10 min at 65°C) and removed by a second purification step with the NucleoSpin RNA Clean-up Kit. 1 µg of RNA was used for first-strand cDNA synthesis with RevertAid Reverse Transcriptase (Thermo Scientific) according to the manufacturer’s instructions, with lugArev as the gene-specific primer. The generated cDNA was then amplified via PCR using lugArev and lugRfwd, which bind in the lugR gene locus. As a control, to exclude genomic DNA contamination, the same PCR was performed on the isolated RNA without previous cDNA synthesis.

### Construction of the YFP reporter system and His-tagged regulators

For the construction of the plasmid-based YFP reporter system, genomic DNA of *S. lugdunensis* IVK28 was isolated using the NucleoSpin Microbial DNA Kit (Macherey & Nagel, Germany). To obtain the different DNA fragments of promoter regions and putative regulators of *lugI, lugJ, lugA*, and *lugR*, genomic DNA was amplified using the primers listed in Table 1. For exchange of the native P*_cap_* promoter located upstream of *yfp* in the reporter plasmid pCG725 (derivative of pCG717 [41] with altered ribosomal binding site), the indicated restriction enzymes were used. For the construction of the promoter-less ΔP*_cap_* plasmid, restriction enzymes SphI and SalI were used to delete the P*_cap_* promoter. Subsequently, the digest was treated with Klenow fragment (Thermo Fisher Scientific) to generate blunt ends and religated. All cloning was performed in *E. coli* DC10B, and sequence-verified plasmids were transferred to *S. aureus* PS187 Δ*hsdR* Δ*sauUSI* by electroporation. To generate 6×His-tagged proteins, *lugR* and *lugJ* were amplified using His-lugR for and rev or His-lugJ for and rev primers, respectively. PCR products and the plasmid pET-28a were digested with BamHI and XhoI and subsequently ligated. To extend the plasmid-encoded 6×His-tag to an 8×His-Tag, whole plasmids containing the regulator genes were amplified using 8×His for and 8×His rev primers. Plasmids were assembled using Gibson Assembly Master Mix (New England Biolabs GmbH, Germany) and further used for transformation of *E. coli* DC10B and *E. coli* Rosetta-gami 2(DE3). Plasmids used are listed in Table 1.

### Construction of *lugR* and *lugJ* deletion mutants and complementation

Deletion of *lugR* and *lugJ* was achieved via allelic replacement. To this end, flanking regions of *lugR* were amplified by PCR with primer pairs lugH SacI/lugH Acc65I u and lugA Acc65I d/lugA BglII. Resulting fragments were digested with the indicated restriction enzymes, ligated into the SacI- and BglII-digested shuttle-vector pBASE6 (42), and transferred into *E. coli* DC10B. Flanking regions of *lugJ* were PCR-amplified with primer pairs lugJ K.O. upstream SacI/lugJ K.O. upstream Acc65I and lugJ K.O. downstream Acc65I- lugJ K.O. downstream BglII, digested with the indicated restriction enzymes, and ligated into pBASE6 as described before. The resulting plasmids were confirmed by sequencing, transferred into *S. aureus* PS187 Δ*hsdR* Δ*sauUSI* by electroporation, and subsequently transduced into *S. lugdunensis* IVK28 as previously described (43). Homologous recombination was achieved as published earlier (42).

To complement the constructed regulator mutants, *lugR* and *lugJ*, including their native promoters, were PCR-amplified from *S. lugdunensis* IVK28 genomic DNA using primers lugR comp_up/lugR comp_do or lugJ comp_up/lugJ comp_do (Table 1), respectively. After digestion with PstI and KpnI (Thermo Scientific), fragments were cloned into the equally digested plasmid pRB473. The ligation mixture was used to transform *E. coli* DC10B. The resulting plasmids (pRB473-lugR and pRB473-lugJ) were verified by PCR and sequencing. Finally, correct complementation plasmids were used for transformation of *S. aureus* PS187∆∆ and subsequent transduction of either *S. lugdunensis* IVK28 ∆*lugR* or *S. lugdunensis* ∆*lugJ* as described above.

### YFP reporter assay

To determine promotor activities of the lugdunin biosynthesis and immunity/transporter operons, YFP expression was measured. Strains were grown overnight in Mueller–Hinton broth (Carl Roth GmbH, Germany) with chloramphenicol. Each strain was adjusted to an optical density at 600 nm (OD_600_) of 1 in BM. 500 µL BM with chloramphenicol and 2 µg/mL lugdunin, if required, were added to a 48-well microtiter plate. Each well was inoculated with 2.5 µL of the OD-adjusted bacterial stock solution. The plates were incubated at 37°C with orbital shaking in a Spark multimode plate reader (Tecan Trading AG). OD_600_ and YFP fluorescence (λ_ex_485 nm, λ_em_535 nm, gain 60) were measured after 24 h. Relative fluorescence units (rel. fl.) were determined after 24 h and normalized to the respective OD_600_.

### Expression and purification of 8×His-LugR and 8×His-LugJ

For the expression and purification of 8xHis-tagged regulators, LugR and LugJ, *E. coli* Rosetta-gami 2(DE3) pET-28a-8x*his-lugR* and pET-28a-8x*his-lugJ* were grown overnight in LB medium with kanamycin. Main cultures (1 L medium in 5 L baffled Erlenmeyer flasks) were inoculated to an OD_600_ of 0.1 and incubated at 37°C with shaking at 130 rpm until an OD_600_ of 0.7 was reached. Protein expression was induced by the addition of 1 mM isopropyl-D-1-thiogalactopyranoside (IPTG) and the cells subsequently cultured at 20°C for 16 h. Cells were harvested by centrifugation at 5,000 × *g* for 20 min at 4°C and washed once with phosphate-buffered saline (pH 7.4). Pellets were resuspended in lysis buffer (20 mM sodium phosphate, pH 7.4, 0.5 M NaCl, 40 mM imidazole, 100 µg/mL lysozyme, one cOmplete^™^ EDTA-free proteinase inhibition cocktail tablet) to a final OD_600_ of 10. After incubation on ice for 30 min, cells were disrupted by sonication (4 × 2 min [3 s on, 5 sc OFF intervals, 40% amplitude]) on ice. Cell lysate was centrifuged at 9,000 × *g* for 30 min at 4°C, and the supernatant was filter sterilized (0.22 µm pore size).

For the subsequent protein purification, which was entirely performed at 4°C, the supernatant was loaded onto columns containing 1 mL charged Ni^2+^ resin (Profinity IMAC resin; Bio-Rad) via gravity flow. The resin was successively washed with buffer containing no imidazole (20 mM sodium phosphate, pH 7.4, 0.5 M NaCl) and a low concentration of imidazole (20 mM sodium phosphate, pH 7.4, 0.5 M NaCl, 20 mM imidazole); at least six column volumes (CVs) of each buffer were used to wash the resin. To elute the His-tagged proteins, the resin was incubated on the column with elution buffer (20 mM ammonium acetate, pH 5.5, 50 mM ammonium sulfate, 10 mM arginine, 200 mM imidazole) for 1 h. Afterwards, the protein containing eluate was collected in falcon tubes. To remove the imidazole for subsequent experiments, a buffer exchange was performed. Therefore, the elution fraction was loaded into Slide-A-Lyzer G3 dialysis cassettes (3.5 kDa MWCO; Thermo Fisher Scientific) and dialyzed against 2× 5 L elution buffer containing no imidazole for 4–6 h and overnight. Protein concentration was determined fluorometrically via Qubit (Qubit 3 fluorometer; Invitrogen). 8×His-LugJ was 3.5-fold concentrated by lyophilization (Alpha 2-4 LDPlus; Martin Christ) and subsequently resuspended in the appropriate volume MiliQ-H_2_0. 8×His-LugR and 8×His-LugJ were stored in 20 µL aliquots at −20°C to avoid freeze–thaw cycles.

### SDS-PAGE and western blot analysis

For denaturing protein analysis, 20 µL samples were mixed with 4 µL 6× Laemmli sample buffer (Bio-Rad Laboratories) and incubated at 95°C for 5 min. Denatured samples were loaded onto a 12% Mini-Protean TGX gel (Bio-Rad Laboratories) and subjected to electrophoresis for 1 h at 120 V. After transfer of the proteins onto a nitrocellulose membrane, proteins were first probed with penta-His antibody (Qiagen, Germany). As the secondary antibody, IRDye 680RD goat anti-rabbit IgG (LI-COR Biosciences) was used. Proteins were visualized using the Odyssey CLx (LI-COR Biosciences, USA).

### Butanol extraction

Overnight cultures of the *S. lugdunensis* IVK28 wild type or Δ*lugD* mutant were used to inoculate BM 1:1,000. The cultures were incubated under constant shaking at 37°C for 24 h. After incubation, the whole cultures were extracted with 1-butanol at a ratio of 3:1 for 1 h. Subsequently, the culture–butanol mixture was centrifuged at 5,000 × g for 30 min, and the upper butanol phase was evaporated at 42°C under reduced pressure.

### Microfractionation of *S. lugdunensis* IVK28 wild type and Δ*lugD* mutant

Butanol extracts with a set concentration of 10 mg/mL were microfractionated by HPLC (Agilent 1260 Infinity II). HPLC-grade water and HPLC-grade acetonitrile were used. HPLC was performed with a gradient from 10 to 100% acetonitrile over 20 min, and the flow rate was set to 1 mL/min. A Kromasil 100 C18 column with a length of 250 mm, an inner diameter of 4 mm, a pore size of 100 A, and a particle size of 5 µM was used.

### Quantification of lugdunin in samples using HPLC–MS

A lugdunin calibration curve was created by solving purified synthetic lugdunin in MeOH at a concentration of 1 mg/mL. The sample was serially diluted at 10-fold steps, and HPLC–MS data were acquired on a Bruker MaXis 4G ESI‐QTOF instrument coupled to a Dionex Ultimate 3000 HPLC system (Thermo Fisher Scientific). Each analysis was calibrated using sodium formate as internal calibrant. The nebulizer pressure of the ESI source was set to 2.0 bar and a dry gas flow rate of 8.0 L/min at an operating temperature of 200°C. For MS–MS spectra acquisition, the auto MS/MS mode with collision energy stepping was used. The mobile phase consisted of solvent A (H_2_O + 0.1% formic acid [FA]) and solvent B (methanol [MeOH] + 0.06% FA). The routine gradient was 10% methanol (0.06% FA) to 100% methanol in 20 min with a flow rate of 0.3 mL/min on a Nucleoshell EC RP‐C18 (150 × 2 mm, 2.7 µm) column from Macherey‐Nagel.

### Electrophoretic mobility shift assay (EMSA)

Promoter DNA fragments of *lugI* (P_lugI_) and *lugR* (P_lugR_) were amplified from IVK28 genomic DNA using primers IR800_lugI_Prom_for/IR800_lugI_Prom_rev or IR800_lugR_Prom_for_/IR800_lugR_Prom_rev, respectively (Table 1; primers contain a 5′ DY-781 label). To generate ‘cold’ promoter fragments with the same sequence but no 5′ label for competitive binding of either LugJ or LugR, the same primers without DY-781 labeling were used.

One nanogram of purified promoter fragments was incubated with appropriate amounts of the regulator proteins (His-LugJ or His-LugR) and lugdunin if required in EMSA buffer (10 mM Tris/HCl, pH 7.4, 10 mM NaCl for LugJ or 100 mM NaCl for LugR, 0.1 mg/mL BSA, 1 mM DTT, 1 mM EDTA, 10% glycerol) and incubated for 30 min at 25°C in a total volume of 20 µl. For specificity evaluation, short DNA fragments containing the putative palindromic recognition sequence were ordered as primers, annealed (heating to 98°C for 5 min and then slow cooling [0.1°C/s] to 10°C), and added to the EMSA reaction. Also, scrambled variants of the palindromes (scram1 and 2) were generated and alternatively added to the EMSA. In the case of scram1, the 5′ half of the palindrome sequence was swapped with the 3′ half. To generate scram2, in the first part of the palindrome, all even bases were swapped with random other bases, while, in the second half, all uneven bases were swapped (methods to generate scram1 and 2 were identical for P*_lugR_* and P*_lugI_* palindromic sequences. Primers are listed in Table 1). For P*_lugR_* binding assays, 5 ng Sau3AI-digested genomic DNA of *S. lugdunensis* 40-2, which is a strain lacking the entire lugdunin operon, was added as non-competitive DNA to improve the running behavior of the His-LugR/P*_lugR_* complex in the gel. Native PAGE gels (5%; 1 mL 40% acrylamide [29:1], 400 µL 1 M Tris-HCl, pH 7.5, 1.5 mL 1 M glycine, 32 µL 0.5 M EDTA, 5.2 mL ddH_2_O, 40 µL 10% ammonium persulfate, 6 µL N,N,N′,N′-trimethylethylenediamine) were pre-run in Tris–glycine buffer (25 mM Tris-HCl, pH 8.3, 193 mM glycine) for 30 min at 100 V. 10 µL of each sample was loaded per lane and subjected to electrophoresis at 100 V for 45–60 min. Subsequently, the native PAGE gels were analyzed and visualized using Odyssey CLx (LI-COR Biosciences, USA).

### Size exclusion chromatography coupled to multi-angle light scattering (SEC–MALS)

SEC–MALS was carried out using a Micro-Äkta chromatography system (GE Healthcare) with a 24 mL Superose 6 Increase 10/300 GL column (GE Healthcare). For MALS analysis, an Optilab T-rEX refractometer and a miniDAWN TREOS detector (Wyatt Technology) were attached to the Micro-Äkta system for accurate molar mass determination. The runs were performed as described before (44). The flow rate was set to 0.5 mL/min, and after equilibration of the column with running buffer (20 mM sodium phosphate, pH 7.4, 0.5 M NaCl), 100 µL of purified 8×His-LugR or 8×His-LugJ, with each protein concentrated to 0.5 mg/mL, was applied. Peak fractions were collected for precipitation with trichloroacetic acid (TCA) and used later for western blot analysis. The elution volume was plotted against the UV signal at 280 nm and against the molecular masses derived from the MALS data. Data analysis and molecular weight calculations were done using ASTRA software (Wyatt). Trimeric His-SbtB with a molar mass of approximately 40 kDa was used as a positive control (45).

### TCA precipitation

Samples from SEC–MALS were mixed with 30% TCA in a 3:1 ratio (sample:TCA) and incubated on ice for 30 min. Afterwards, samples were centrifuged at 20,000 × *g* for 15 min at 4°C. After removal of the supernatant, the pellet was washed three times with 80% acetone, dried for 10 min at 95°C, and finally resuspended in 20 µL water.

### Molecular modeling

LugR and LugJ dimeric models were generated using AlphaFold, and all lugdunin isomers were docked into predicted binding sites. The proposed binding mode systems were then simulated in an all-atom and explicit solvent system for several microseconds and had their protein–ligand interaction frequencies and binding energy variation monitored along the simulated trajectory (see supplemental material methods for the entire pipeline description).

## Data Availability

The genomic data supporting the findings of this study can be retrieved from the National Center for Biotechnology Information (NCBI) with the *S. lugdunensis* IVK28 genome sequence (GenBank accession number CP063143) and LugR (WP_002460032) and LugJ (WP_002460039) protein sequences. All molecular dynamics trajectories and raw data related to the protein-ligand interactions within the simulations are available at https://zenodo.org/records/7648534 (repository: DOI 10.5281/zenodo.7648534).

## SUPPLEMENTAL MATERIAL

Supplemental material mbio.03571-24-s0001.pdf Supplemental figures and text.

ASM does not own the copyrights to Supplemental Material that may be linked to, or accessed through, an article. The authors have granted ASM a non-exclusive, world-wide license to publish the Supplemental Material files. Please contact the corresponding author directly for reuse.
