# Supplementary material for: The human microbiome-derived antimicrobial lugdunin self-regulates its biosynthesis by a feed-forward mechanism
Source: mBio. 2025 Mar 18;16(4):e03571-24. doi: 10.1128/mbio.03571-24 (PMC11980582; doi:10.1128/mbio.03571-24)
Supplement: Supplemental material — Supplemental figures and text. [file mbio.03571-24-s0001.pdf]

Supplementary Figures

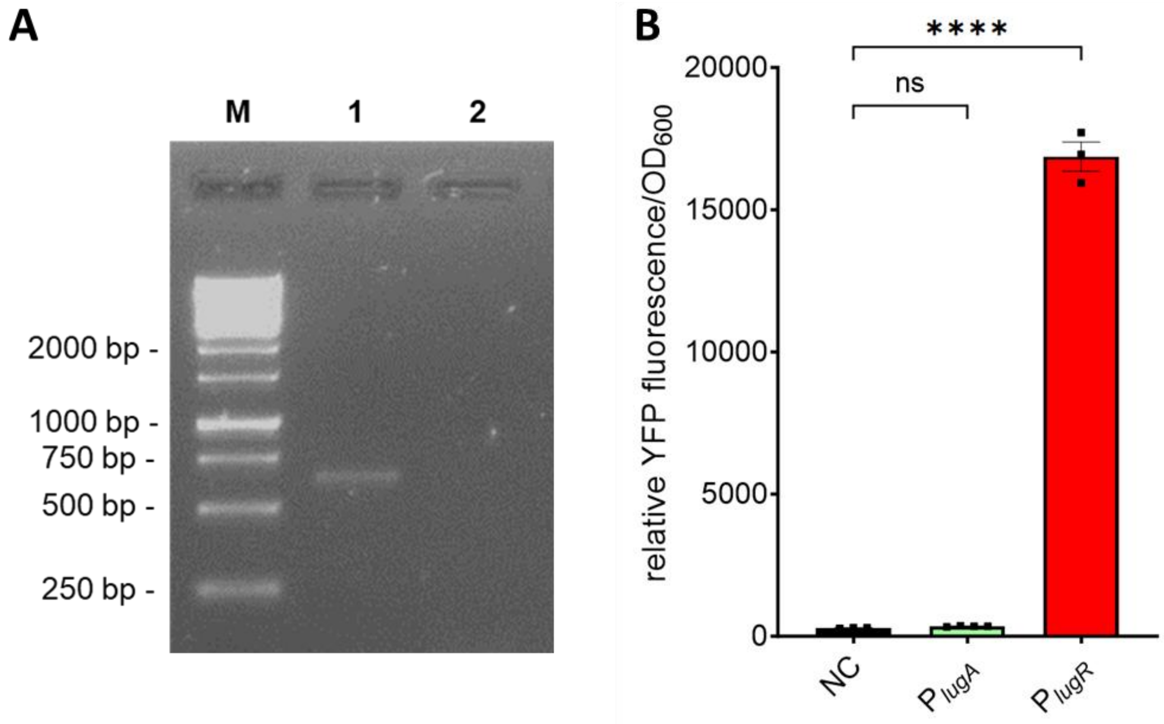

Figure S1. RT-PCR of the *lugRA* region and promoter activity testing of P_{lugA} and P_{lugJ}. A) RT-PCR of *lugRA* with cDNA (1) or RNA (2) as template (DNAseI treatment of RNA before reverse transcription). Only cDNA allows amplification of a 660 bp fragment confirming the location of *lugR* and *lugA* on a single transcript. B) Endpoint measurements of YFP fluorescence in *S. aureus* PS187 strains carrying the reporter construct P_{lugA} or P_{lugR}. The measured YFP intensities were correlated to the respective OD_{600} after 24 h of growth at 37°C. M: DNA size marker, NC; negative control. Data represent mean values ± SEM of three independent biological replicates. All data were analysed using One-Way ANOVA, ns=not significant, **** p<0.0001.

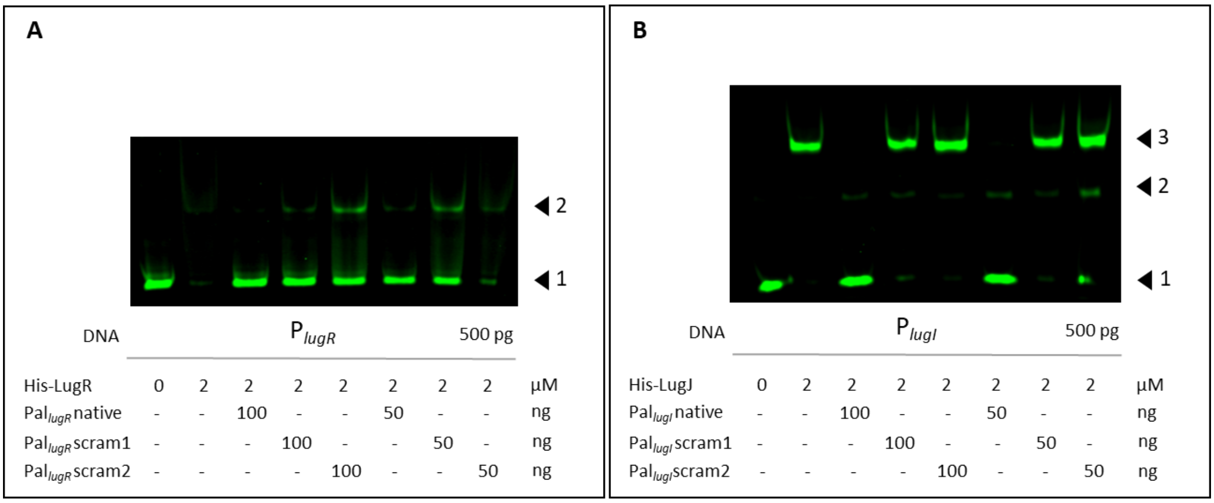

Figure S2. Palindrome sequence specificity of the lugdunin regulators LugR and LugJ

DY-781 labelled DNA-promoter fragments, regulator proteins and specific or scrambled palindrome sequences were incubated in EMSA buffer and analysed via native-PAGE. 500 pg of the DY-781 labelled P_{lugI} or P_{lugR} PCR fragment was used for interaction studies with His-LugJ or His-LugR, respectively. A) 2µM of His-LugR was used for the EMSA with labelled P_{lugR} promoter fragment, leading to a shift of P_{lugR}. Addition of 100 or 50 ng of the predicted native palindromic recognition sequence of LugR (Pal_{lugR} native), led to an abrogation of the shift. Addition of the same amount of DNA containing scrambled versions of the palindrome (Pal_{lugR} scram1,2) were less effective to release the shift. B) 2µM of His-LugJ was used for the EMSA with labelled P_{lugI} promoter fragment, leading to a shift of P_{lugI}. Addition of 100 or 50 ng of DNA, containing the predicted native palindromic recognition sequence of LugJ (Pal_{lugI} native), led to the complete displacement of the fluorescently labeled promoter fragment. In contrast, addition of the same amount of DNA containing scrambled versions of the palindrome (Pal_{lugI} scram1,2) had no influence on the shift. Black arrows indicate the positions of the shifted Promoter fragments (1: unbound promoter fragment; 2: shifted DNA-protein complex; 3: double shifted DNA-protein complex).

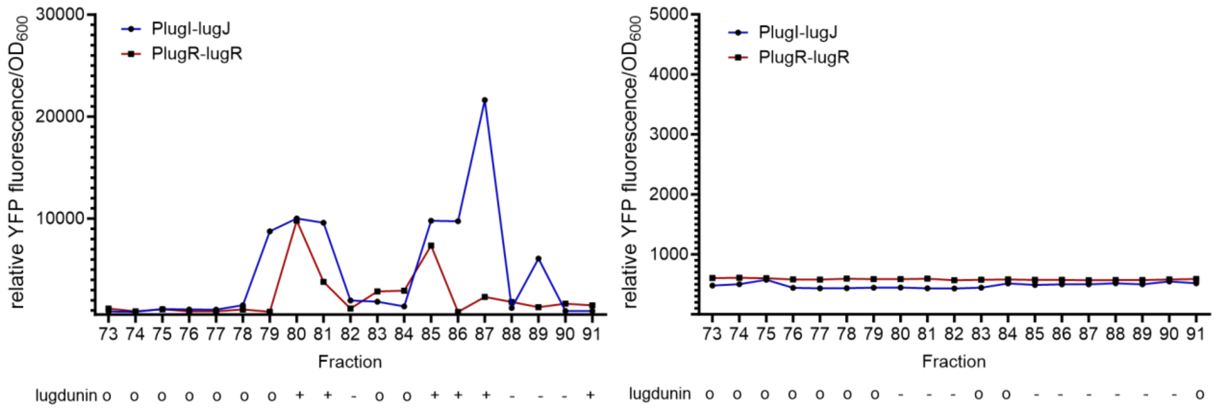

Figure S3. YFP signal intensity of *S. aureus* PS187 pCG725-P$_{lugI}$-*lugJ* or pCG725-P$_{lugR}$-*lugR* in response to components from *S. lugdunensis* IVK28 superatant. An overnight culture of (A) *S. lugdunensis* IVK28 or (B) *S. lugdunensis* IVK28 Δ*lugD* was 1-butanol extracted and microfractionated in a 96-well plate. The samples were dried and reconstituted with BM media containing (red) *S. aureus* PS187 ΔΔ pCG725-P$_{lugI}$-*lugJ* or (blue) *S. aureus* PS187 ΔΔ pCG725-P$_{lugR}$-*lugR.* Relative fluorescence intensities and optical densities were determined after 24 h. Fractions were then analysed for the presence of lugdunin with HR-MS, o = not analysed, - = no lugdunin identified, + = lugdunin identified.

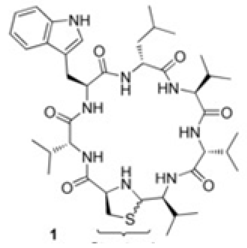

1

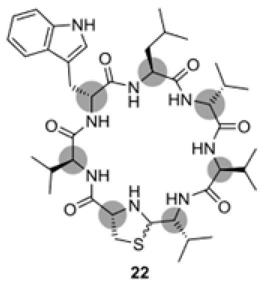

2

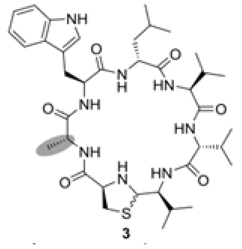

3

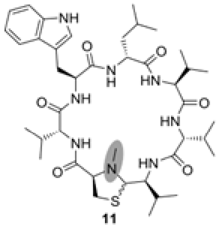

4

Figure S4. Chemical structures of lugdunin and its derivatives. Lugdunin (1), enantio-lugdunin (2), 2-Ala-lugdunin (3) and N-methylthiazolidine-lugdunin (4).

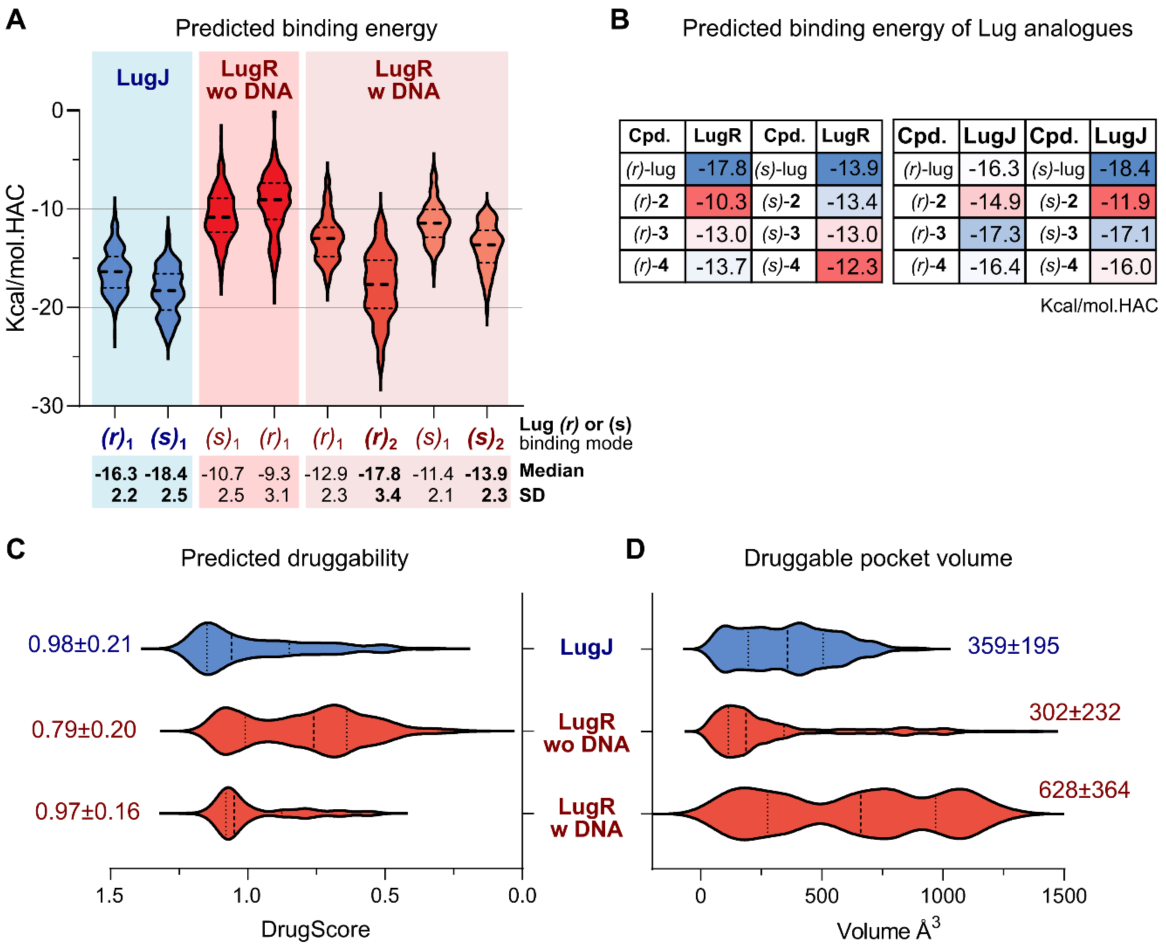

Cpd.	LugR	Cpd.	LugR
(r)-lug	-17.8	*(s)*-lug	-13.9
(r)-**2**	-10.3	*(s)*-**2**	-13.4
(r)-**3**	-13.0	*(s)*-**3**	-13.0
(r)-**4**	-13.7	*(s)*-**4**	-12.3

Cpd.	LugJ	Cpd.	LugJ
(r)-lug	-16.3	*(s)*-lug	-18.4
(r)-**2**	-14.9	*(s)*-**2**	-11.9
(r)-**3**	-17.3	*(s)*-**3**	-17.1
(r)-**4**	-16.4	*(s)*-**4**	-16.0

Figure S5. Characterization of the predicted binding pockets of LugJ and LugR. A) ligand efficiency prediction distribution for the simulations displayed as violin plots. Ligand efficiency was calculated using MM-GBSA's predicted binding energy (see methods) and is represented by its mean + standard deviation. HAC: heavy atom count; r: (*r*)-lugdunin (R-lug; also represented by dashed boxes) and s: (*s*)-lugdunin (S-lug; plain boxes), numbers represent potential binding modes generated as alternative solutions results of docking. B) single point binding energy calculations for distinct lugdunin analogues within the cluster-representative binding pocket for each transcription factor. Predicted druggable binding pocket monitored along the simulation time for LugJ and LugR models (with and without DNA), in terms of predicted druggability (DrugScores, C) and volume (D, volume is expressed in cubic Angstroms), with their respective median and standard deviation values. Calculated free-binding energies are represented by the MM/GBSA and normalized by the number of heavy atoms (HAC), according to the following formula: ligand efficiency = (energy) / (1 + ln(HAC)).

Supplementary information

Molecular modelling.

Homology model and protein preparation. The *S. lugdunensis* IVK28's LugJ (accession number: WP_002460039) and LugR (WP_002460032) monomeric homology models were generated using the ColabFold (1) implementation of the AlphaFold (2) with standard options.

LugJ showed an HTH-containing regulator folding and its multimeric structure was modelled after the (PDB ID: 4G6Q, resolution: 2.1 Å, a transcription factor from *K. flavida*, unpublished but deposited). The DNA would bind in the lower helix-wing portion of the template's N-termini, similar to the MarR family (3). However, the absence of the typical arginine patch in the potential DNA surface (blocked by the dimerization interface) did not allow us to place a nucleic acid chain by superimposition. Therefore, we proceed with a final model without dsDNA.

On the other hand, given the high similarity in terms of folding between LudR and the TetR/FadR transcription factor family, as well as the availability of high-quality structural data, we decided to include dsDNA coordinates from FadR (PDB ID: 5GPC, resolution: 2.8 Å), in our final models by superimposing the structures and retrieving their structure. Control LugR models without dsDNA were generated based on the ketosteroid transcriptional regulator of *Mycobacterium tuberculosis* (PDB ID: 4W97, Resolution: 1.60 Å (4)), which was selected due to its high resolution and completeness. Though it would sound logical to generate our LugR working model after the inhibited state, its binding pocket was too constricted to generate relevant binding mode poses, despite the similar drugability potential (Supporting information, Figure S5C,D).

Final models were validated by checking its Ramachandran plot and overall energy levels, low confidence level regions in the N- and C-termini (as already suggested by the ColabFold scores) indeed displayed higher energy and underwent energy minimization as a last step of preparation. All protein structures were prepared using the Protein Wizard Preparation tool (Schrödinger LCC), with standard options and the homology model was further refined to remove sterical clashes. For all structures protonation states of amino acids were optimized with PROPKA (Schrödinger, LLC, New York, NY, 2022.4), where we selected the most likely ionization state as proposed by the software, and the structures were minimized using steep descent (cut-off for heavy atoms 1 Å).

Ligand preparation and molecular docking. Three-dimensional ligand structures were generated with LigPrep, using Epik (5) to predict their protonation in pH 7.0 ± 1.0. The diastereoisomer configuration was derived from the synthesis (6), except for the thiazolidine ring, which was simulated for both, (*r*)-lugdunin (herein R-lug) and (*s*)-lugdunin (S-lug) configurations. The OPLS4 force field (7) was employed for structure generation. Each prepared ligand underwent extensive conformer generation (using MacroModel with implicit solvent and standard options), and all conformers were submitted to docking.

Ligands were docked within a grid around 13 Å from the centroid of the potential binding pocket, identified using SiteMap2 (Schrödinger LCC). Docking was performed using Glide (8,9) using the SiteMap-identified pocket and employing XP scoring function. All docking poses were visually inspected, independently from the docking score, and those with the highest number of consistent interactions were selected for simulation.

Molecular dynamics (MD) simulations. MD simulations were carried out by using the Desmond engine (10) with the OPLS4 force-field (7). The system encompassed the protein-ligand/cofactor complex, a predefined water model (TIP3P) (11) as a solvent and counterions (Na^+ or Cl^- adjusted to neutralize the overall system charge). The system was treated in a cubic box with a periodic boundary condition (PBC) specifying the shape and the size of the box as 13 Å distance from the box edges to any atom of the protein. Short-range coulombic interactions were calculated using 1-fs time steps and 9.0-Å cut-off value, whereas long-range coulombic interactions were estimated using the Smooth Particle Mesh Ewald (PME) method (12,13).

Initially, the relaxation of the system was performed using Steepest Descent and the limited-memory Broyden-Fletcher-Goldfarb-Shanno algorithms in a hybrid manner, according to the established protocol available in the Desmond standard settings. During the equilibration step, the simulation was performed under the NPT ensemble for 5 ns implementing the Berendsen thermostat and barostat methods (14). A constant temperature of 310 K was kept throughout the simulation using the Nose-Hoover thermostat algorithm (15) and Martyna-Tobias-Klein Barostat algorithm (16) to maintain 1 atm of pressure, respectively.

After minimization and relaxation of the system, each protein+lugdunin system was subjected to at least 1-μs simulations (five replicas of 200 ns each) with random seeds, unless stated otherwise. All the trajectories, interaction data, and RMSD/RMSF reports are available on the Zenodo repository (code: 10.5281/zenodo.7648534). MD trajectories were visualized, and figures were generated using PyMOL v.2.5.2 (Schrödinger LCC, New York, NY, USA).

MD simulation trajectory analyses. Protein-ligand interactions and atomic distances were calculated using the Simulation Event Analysis pipeline as implemented in Maestro (v2022.4, Schrödinger LCC) using standard options. Representative frames of the simulations were retrieved using hierarchical clustering analyses. Trajectories were clustered using the script trj_cluster.py (implemented in Maestro 2022.4, Schrödinger LCC) according to the RMSD of ligand's heavy atoms, using 1 Å as the cut-off. RMSD values of the protein backbone were used to monitor simulation equilibration and protein folding changes (all raw data is available in the repository link).

MM-GBSA binding energy calculations. Molecular mechanics with generalized Born and surface area (MM-GBSA) predicts the binding free energy of protein-ligand complexes and the ranking of ligands based on the free energy could be correlated to the experimental binding affinities, especially in a congeneric series. Every 10th frame from the simulations was considered for the calculations. These were used as input files for the MM-GBSA calculations with thermal_mmgbsa.py script from the Schrödinger package. Calculated free-binding energies are represented by the MM/GBSA and normalized by the number of heavy atoms (HAC), according to the following formula: ligand efficiency = ln(docking score) / (1 + ln(HAC)).

Pocket volume and druggability scores were calculated using SiteMap along the trajectory with the script trajectory_binding_site_volumes.py, calculating every 10th frame from the simulations.

Supplementary References

1. Mirdita, M., Schütze, K., Moriwaki, Y., Heo, L., Ovchinnikov, S., and Steinegger, M. (2022). ColabFold: making protein folding accessible to all. Nature Methods *19*, 679-+. 10.1038/s41592-022-01488-1.
2. Jumper, J., Evans, R., Pritzel, A., Green, T., Figurnov, M., Ronneberger, O., Tunyasuvunakool, K., Bates, R., Zídek, A., Potapenko, A., et al. (2021). Highly accurate protein structure prediction with AlphaFold. Nature *596*, 583-+. 10.1038/s41586-021-03819-2.
3. Corbella, M., Liao, Q.H., Moreira, C., Parracino, A., Kasson, P.M., and Kamerlin, S.C.L. (2021). The N-terminal Helix-Turn-Helix Motif of Transcription Factors MarA and Rob Drives DNA Recognition. J Phys Chem B *125*, 6791-6806. 10.1021/acs.jpcb.1c00771.
4. Crowe, A.M., Stogios, P.J., Casabon, I., Evdokimova, E., Savchenko, A., and Eltis, L.D. (2015). Structural and Functional Characterization of a Ketosteroid Transcriptional Regulator of. Journal of Biological Chemistry *290*, 872-882. 10.1074/jbc.M114.607481.
5. Shelley, J.C., Cholleti, A., Frye, L.L., Greenwood, J.R., Timlin, M.R., and Uchimaya, M. (2007). Epik:: a software program for pK prediction and protonation state generation for drug-like molecules. J Comput Aid Mol Des *21*, 681-691. 10.1007/s10822-007-9133-z.
6. Schilling, N.A., Berscheid, A., Schumacher, J., Saur, J.S., Konnerth, M.C., Wirtz, S.N., Beltrán-Beleña, J.M., Zipperer, A., Krismer, B., Peschel, A., et al. (2019). Synthetic Lugdunin Analogues Reveal Essential Structural Motifs for Antimicrobial Action and Proton Translocation Capability. Angew Chem Int Edit *58*, 9234-9238. 10.1002/anie.201901589.
7. Lu, C., Wu, C.J., Ghoreishi, D., Chen, W., Wang, L.L., Damm, W., Ross, G.A., Dahlgren, M.K., Russell, E., Von Bargen, C.D., et al. (2021). OPLS4: Improving Force Field Accuracy on Challenging Regimes of Chemical Space. J Chem Theory Comput *17*, 4291-4300. 10.1021/acs.jctc.1c00302.
8. Friesner, R.A., Banks, J.L., Murphy, R.B., Halgren, T.A., Klicic, J.J., Mainz, D.T., Repasky, M.P., Knoll, E.H., Shelley, M., Perry, J.K., et al. (2004). Glide: A new approach for rapid, accurate docking and scoring. 1. Method and assessment of docking accuracy. Journal of Medicinal Chemistry *47*, 1739-1749. 10.1021/jm0306430.
9. Friesner, R.A., Murphy, R.B., Repasky, M.P., Frye, L.L., Greenwood, J.R., Halgren, T.A., Sanschagrin, P.C., and Mainz, D.T. (2006). Extra precision glide: Docking and scoring incorporating a model of hydrophobic enclosure for protein-ligand complexes. Journal of Medicinal Chemistry *49*, 6177-6196. 10.1021/jm051256o.
10. Bowers, K.J., Chow, E., Xu, H., Dror, R.O., Eastwood, M.P., Gregersen, B.A., Klepeis, J.L., Kolossvary, I., Moraes, M.A., Sacerdoti, F.D., et al. (2006). Scalable algorithms for molecular dynamics simulations on commodity clusters. Proceedings of the 2006 ACM/IEEE conference on Supercomputing. Association for Computing Machinery.
11. Jorgensen, W.L., Chandrasekhar, J., Madura, J.D., Impey, R.W., and Klein, M.L. (1983). Comparison of Simple Potential Functions for Simulating Liquid Water. J Chem Phys *79*, 926-935. Doi 10.1063/1.445869.
12. Darden, T., York, D., and Pedersen, L. (1993). Particle Mesh Ewald - an N.Log(N) Method for Ewald Sums in Large Systems. J Chem Phys *98*, 10089-10092. Doi 10.1063/1.464397.
13. Cheatham, T.E., Miller, J.L., Fox, T., Darden, T.A., and Kollman, P.A. (1995). Molecular-Dynamics Simulations on Solvated Biomolecular Systems - the Particle Mesh Ewald Method Leads to Stable Trajectories of DNA, Rna, and Proteins. Journal of the American Chemical Society *117*, 4193-4194. DOI 10.1021/ja00119a045.

14. Berendsen, H.J.C., Postma, J.P.M., Vangunsteren, W.F., Dinola, A., and Haak, J.R. (1984). Molecular-Dynamics with Coupling to an External Bath. J Chem Phys *81*, 3684-3690. Doi 10.1063/1.448118.
15. Martyna, G.J., Klein, M.L., and Tuckerman, M. (1992). Nose-Hoover Chains - the Canonical Ensemble Via Continuous Dynamics. J Chem Phys *97*, 2635-2643. Doi 10.1063/1.463940.
16. Martyna, G.J., Tuckerman, M.E., Tobias, D.J., and Klein, M.L. (1996). Explicit reversible integrators for extended systems dynamics. Mol Phys *87*, 1117-1157. Doi 10.1080/00268979600100761.
